# The complete chloroplast genome of *Syringa oblata* (Oleaceae)

**DOI:** 10.1080/23802359.2020.1772143

**Published:** 2020-06-02

**Authors:** Mingde Zhao, Yongmei Zhang, Zhiying Xin, Xianwen Meng, Wenying Wang

**Affiliations:** a College of Ecological Environment and Resources, Qinghai Nationalities University, Xining, China; b Division of science and technology, Qinghai Normal University, Xining, China

**Keywords:** *Syringa oblata*, Oleaceae, chloroplast genome, phylogenetic tree

## Abstract

*Syringa oblata* Lindl. is a popular ornamental shrub with aroma compounds. Here, we sequenced and assembled the complete chloroplast genome of *S. oblata*. The complete chloroplast genome of *S. oblata* is 155,648 bp in length, containing a pair of inverted repeated (IRa and IRb) region of 25,732 bp that are separated by a large single copy (LSC) region of 86,247 bp, and a small single copy (SSC) region of 17,937 bp. A total of 132 functional genes were annotated, including 88 protein-coding genes, 36 tRNA genes, and eight rRNA genes. The Neighbour-joining phylogenetic tree based on complete chloroplast genomes suggested that *S. oblata* is most closely related to *S. vulgaris*.

*Syringa* (Lilac) is among the popular ornamental bushes and is widely cultivated in the northern hemisphere. Flowers of *Syringa oblata* Lindl. bloom from April to May in China. This flower releases the aroma compounds, which are very pleasant to the human sensory system (Li et al. 2006). Chloroplast genome sequences are the most important source of genetic markers to study the distribution of paternal genes and paternally based molecular phylogenetic relationships (Shaw et al. 2007). In this study, the complete chloroplast genome (accession number: MT025818) of *S. oblata* was *de novo* sequenced based on new generation sequencing (NGS) technology.

The samples of *S. oblata* were collected from Xining Botanical Garden, Xining, China (36.60°N, 101.76°E). The experiment and analysis scheme refers to Wang et al. (2019). The experiment and analysis scheme also refer to Wang et al. (2019). Total DNA of *S. oblata* was extracted from the dried, young leaves (about 0.3 g) with a modified CTAB method (Doyle and Doyle 1987). The voucher specimen (Specimen Accession number: WJL2019035) was kept in Herbarium of the Northwest Institute of Plateau Biology, Chinese Academy of Sciences (HNWP). Genome sequencing was performed using the Illumina HiSeq Platform (Illumina, San Diego, CA) at Genepioneer Biotechnologies Inc., Nanjing, China. Approximately 6.13 GB of clean data was yielded. The trimmed reads were mainly assembled by SPAdes (Bankevich et al. 2012). The assembled genome was annotated using CpGAVAS (Liu et al. 2012).

The complete chloroplast genome of *S. oblata* is 155,648 bp in length, containing a pair of inverted repeated (IRa and IRb) region of 25,732 bp that are separated by a large single copy (LSC) region of 86,247 bp, and a small single copy (SSC) region of 17,937 bp. A total of 132 functional genes were annotated, including 88 protein-coding genes, 36 tRNA genes, and eight rRNA genes. The protein-coding genes, tRNA genes, and rRNA genes account for 66.67%, 27.27%, and 6.06% of all annotated functional genes, respectively.

Phylogenetic relationships of *S. oblata* and its closely related species were resolved by means of Neighbour-joining (NJ). The alignment was conducted using MAFFT (Katoh and Standley 2013; online version: https://mafft.cbrc.jp/alignment/server/). The Neighbour-Joining (NJ) tree was constructed using the MEGA7 package (Kumar et al. 2016) with 1000 bootstrap repetitions. The NJ phylogenetic tree suggested that *S. oblata* is most closely related to *S. vulgaris* (Figure 1).

**Figure 1.**
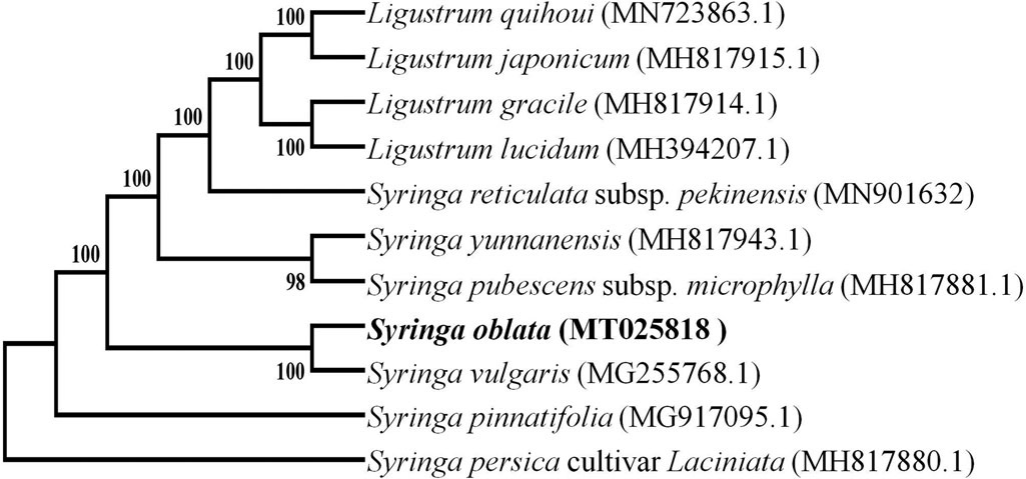
The NJ tree based on chloroplast genome sequences.

## Data Availability

The data that support the findings of this study are openly available in Genbank at https://www.ncbi.nlm.nih.gov/genbank/, reference number MT025818.
